# Evaluation of novel SexedULTRA-4M technology for *in vitro* bovine embryo production

**DOI:** 10.1590/1984-3143-AR2022-0018

**Published:** 2022-04-22

**Authors:** Horacio Álvarez-Gallardo, Michael Edward Kjelland, Mario Pérez-Martínez, Fernando Villaseñor-González, Salvador Romo-García

**Affiliations:** 1 Facultad de Estudios Superiores Cuautitlán, Universidad Nacional Autónoma de México, Cuautitlán, México, México; 2 Centro Nacional de Recursos Genéticos, Instituto Nacional de Investigaciones Forestales, Agrícolas y Pecuarias, Tepatitlán, Jalisco, México; 3 Conservation, Genetics & Biotech, LLC, Valley City, North Dakota, USA; 4 Mayville State University, North Dakota, USA; 5 Facultad de Medicina Veterinaria y Zootecnia, Universidad Nacional Autónoma de México, CDMX, México; 6 Campo Experimental Centro Altos de Jalisco, Instituto Nacional de Investigaciones Forestales, Agrícolas y Pecuarias, Tepatitlán, Jalisco, México

**Keywords:** SexedULTRA-4M, sexed semen, bovine, IVF

## Abstract

SexedULTRA-4M™ is made using an improved method of sex-sorting sperm in a less damaging environment for better retaining sperm integrity throughout the sorting process. The objective of this research was to compare conventional (CONV) and SexedULTRA-4M™ (ULTRA-4M) semen for bovine IVP using four Angus bulls. Matured slaughterhouse oocytes (n = 4000) were divided into the CONV group and the ULTRA-4M group (2000 COCs for each semen type). The IVF process was implemented with CONV and ULTRA-4M semen from the same bull. The cleavage rates, eight cell embryos and blastocysts on day 7 of culture were evaluated for each semen type and each bull. The statistical analysis was carried out with the ANOVA procedure SAS software. The results were 54.45% ± 1.03 and 58.10% ± 1.07; 35% ± 1.57 and 39.15% ± 1.62; 22.8% ± 1.09 and 27.15% ± 1.12 for CONV and ULTRA-4M, respectively, for cleavage rate, eight cell embryos and blastocysts on day 7 for the average of all bulls, comparing only the semen type. Concerning only the semen type, ULTRA-4M was significantly superior to CONV for cleavage rates (P = 0.01) and blastocysts on day 7 (P = 0.009). There were no significant differences between the CONV and ULTRA-4M groups (P>0.05) for all variables analyzed for Bull 1 and Bull 4, however, for Bull 2 ULTRA-4M was significantly superior to CONV for cleavage rates and blastocysts on day 7 (P< 0.05). In Bull 3, ULTRA-4M was significantly higher (P< 0.05) for blastocysts on day 7 compared to CONV. In conclusion, under the conditions of this research the ULTRA-4M and CONV semen produced similar bovine IVP results overall.

## Introduction

For thousands of years, livestock owners have desired a methodology to predetermine the sex of the offspring of their herds. For dairy cattle, this means heifer calves are often the most desirable sex given milk production (Wheeler et al.*,* 2006). Sex ratio of the resulting progeny through natural mating or through an artificial breeding program is genetically controlled, but the one disadvantage is the fixed probability of 51:49 in favor of male calves. This is one of the few genetic traits that cannot be controlled or manipulated efficiently by breeding programs alone (Seidel, 2003). The first commercial license of sexed semen was granted to Cogent company in the United Kingdom in 2002 (Seidel, 2014). Since then, millions of offspring have been born using sexed semen produced by flow cytometry. Sperm can be successfully sorted based on the DNA content between the X and Y chromosomes, however, this technique reduced the viability and quality of frozen-thawed sex sorted semen through the extended holding time before staining, exposure to a laser beam to induce fluorescence, separation into X- and Y- chromosome-bearing sperm and finally exposure to an electrical field for drafting as a relatively pure population into an appropriate vessel, all of which may contribute to the reduction in fertility, a decrease of about 20–40% compared to that of unsexed spermatozoa (Seidel and Garner, 2002).

Historically, it was always considered that the most economical method to use sex-sorted sperm in breeding programs would be through IVF methods where a relatively small number of spermatozoa are required. The combination of ovum pick-up with sexed semen is a potential tool to generate sexed embryos through IVF (Vishwanath and Moreno, 2018). Sexed semen has been used in several studies to produce embryos *in vitro* (Cran et al., 1993, 1995; Lu et al., 1999; Zhang et al., 2003; Lu and Seidel, 2004; Fischer-Brown et al., 2005; Wilson et al., 2005, 2006; Palma et al., 2008). Many issues appear to influence the success rates when sexed semen is used to produce bovine embryos *in vitro*. These issues include lower fertilization rates (Cran et al.*,* 1995), lower cleavage rates (Lu et al., 1999), lower blastocyst rates (Lu et al., 1999; Merton et al.*,* 1997), lower pregnancy rates (Cran et al., 1995), partial capacitation of the sperm, dilute sperm samples (Lu and Seidel, 2004), and sire variation (Zhang et al., 2003; Barceló-Fimbres et al., 2011; Palma et al., 2008). Currently, the technology of sexed semen has been modernized to what is now known as SexedULTRA-4M™ (ST Genetics), completely modifying the technique, the media, and sperm concentration (Vishwanath and Moreno, 2018). Based on the few studies in the scientific literature, this new sexed semen technology seems to have similar results compared to conventional semen when used for artificial insemination: 66.7 vs 65.6% (Lenz et al., 2017), 52 vs 60% (Thomas et al., 2017), and 52 vs 58% (Thomas et al., 2019), and with 95 to 96% of sex precision (Thomas et al., 2017, 2019). However, the scientific literature lacks information about *in vitro* embryo production (IVP) with X chromosome-bearing SexedULTRA-4M™ semen (Gonzalez-Marin et al., 2017). Hence the objective of this research was to compare the use of conventional (CONV) and SexedULTRA-4M™ (ULTRA-4M) semen to produce bovine embryos *in vitro*.

## Methods

### Oocyte collection and *in vitro* maturation (IVM)

The present manuscript is in accordance with the procedures and regulations and that proper authorization has been received by the Research Ethics Commission of the Institution of origin. All procedures were conducted in accordance with the guidelines provided by the Ethics Committee for Care and Use of Laboratory Animals for Research of the Universidad Nacional Autónoma de México, México (Protocol No: SICUAE.DC-2019/1-1).

Ovaries from commercial cattle were collected from a slaughterhouse (León, México), and transported to the laboratory within 2 h in physiological saline solution (0.9% NaCl) supplemented with penicillin G (100 UI/mL) and streptomycin sulfate (100 µg/mL) at 35 °C. Cumulus-oocyte complexes (COCs) were aspirated from follicles 2-8 mm in diameter using an 18-gauge needle attached to 5 mL disposable syringe. The COCs with evenly granulated cytoplasm and enclosed by more than three layers of compact cumulus cells were selected (only COCs grades 1 and 2), washed three times in wash medium (Vitrogen, Brazil) and twice in IVM medium (Vitrogen, Brazil) and matured *in vitro* in a four well dish (Nunc, Denmark) for 24 h at 38.5 °C in 5% CO_2_ in air and 100% humidity (500 µL / 50 oocytes).

### *In vitro* fertilization (IVF)

COCs were classified as matured oocytes according to the classification of Aguila et al. (2020), only grade 3 (complete expansion) oocytes were used. Matured oocytes (n = 4000, divided equally into five replicates) were divided into two groups, the CONV and the ULTRA-4M. The IVF process was conducted with commercially produced (ST Genetics) CONV and ULTRA-4M (X chromosome-bearing) semen from contemporaneous ejaculates from the same bull each, using four different bulls from the same breed (Angus). After maturation, COCs were washed three times in IVF medium (Vitrogen, Brazil) and placed in 50 µL droplets overlaid with mineral oil (Vitrogen, Brazil) 30 min before the fertilization.

Both semen samples (CONV and ULTRA-4M) were thawed by immersing the straw in a water bath at 37 °C and were selected through Mini-Percoll gradients using two columns of 400 µL each, using conventional and sexed Percoll (Vitrogen, Brazil) for CONV and ULTRA-4M respectively, and centrifugated at 600 *g* for 6 min and washed with 500 µL IVF medium at 600 *g* for 3 min. The pellet was resuspended in IVF medium to give a final concentration of 1 x 10^6^ sperm/mL, for CONV and ULTRA-4M for 18 h in 38.5 °C, 5% CO_2_ in air and 100% humidity.

### *In vitro* culture

After 18 hours from the beginning of *in vitro* fertilization, the presumptive zygotes were denuded by pipetting, washed 5 times in wash medium (Vitrogen, Brazil) to remove the debris and placed in IVC medium (Vitrogen, Brazil) until day 7 at 38.5 °C, 5% CO_2_, 5% O_2_ and 90% N_2_ at 100% humidity. On day 3 (56 h after *in vitro* culture), the cleavage rate and eight cell embryos were recorded and blastocysts on day 7 were evaluated.

### Experimental design

The 4000 collected oocytes were subjected to a 2 X 4 factorial experimental design with two types of semen (CONV and ULTRA-4M), and both types of semen from four bulls (1-4).

### Statistical analysis

Each experiment was replicated 5 times, 100 COCs (pooled) were used for each replicate and for each kind of semen from 4 bulls (4000 COCs). The cleavage rate, eight cell embryos and blastocysts on day 7 were evaluated. The statistical analysis was carried out with the ANOVA procedure of the SAS software (version 9.3; SAS Institute Inc., Cary, NC, USA). A Tukey HSD Post Hoc Test was done to determine the differences between the groups for each bull (1-4) with both semen types (CONV and ULTRA-4M). Differences were considered significant at P<0.05.

## Results

**Figure 1:**
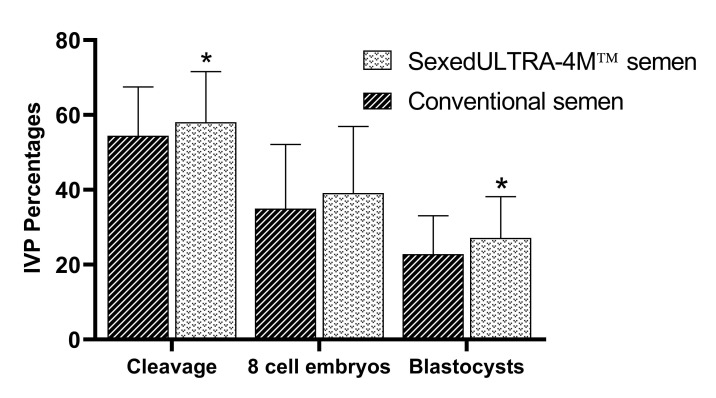
Summary of IVP using conventional and SexedULTRA-4M™ semen. *Bars with superscripts differ (P<0.05).

For the average of the four bulls, the results for CONV were 54.45% ± 1.03, 35% ± 1.57, and 22.8% ± 1.09; and for ULTRA-4M were 58.10% ± 1.07, 39.15% ± 1.62, and 27.15% ± 1.12 respectively, for cleavage rate, eight cell embryos and blastocysts on day 7. ULTRA-4M was significantly superior to CONV for cleavage rates (P = 0.01) and blastocysts on day 7 (P = 0.009), these data are shown in Figure 1.

Evaluating the results of the bulls, cleavage rates were 72.2% ± 5.63 and 75.6% ± 5.73 (P = 0.37); 46.2% ± 2.28 and 51% ± 3.32 (P = 0.029); 40.8% ± 2.28 and 43% ± 2.55 (P = 0.18); 58.6% ± 6.02 and 62.8% ± 7.79 (P = 0.36) respectively in CONV and ULTRA-4M groups for Bull 1, Bull 2, Bull 3, and Bull 4. The percentages of eight cell embryos were 59.8% ± 11.03 and 62.8% ± 13.92 (P = 0.71); 26.2% ± 1.92 and 29.8% ± 3.63 (P = 0.08); 18% ± 1.87 and 20.8% ± 2.58 (P = 0.08); 36% ± 6.48 and 43.2% ± 6.34 (P = 0.11), respectively, with CONV and ULTRA-4M groups with Bull 1, Bull 2, Bull 3, and Bull 4. The percentages of blastocysts on day 7 with Bull 1 for CONV were 35.8% ± 6.42 and 40.4% ± 8.91 (P = 0.37) for ULTRA-4M; with Bull 2 for CONV were 20.4% ± 2.07 and 24.2% ± 2.77 (P = 0.04) for ULTRA-4M; with Bull 3 for CONV were 10.4% ± 1.14 and 14.2% ± 0.83 (P < 0.001) for ULTRA-4M; and with Bull 4 for CONV were 24.6% ± 6.35 and 29.8% ± 6.65 (P = 0.24) for ULTRA-4M (Table 1).

**Table 1:** Results of the *in vitro* embryo production of bovine embryos using CONV and ULTRA-4M semen from four bulls.

**BULL**	**SEMEN**	**OOCYTES N**	**CLEAVAGE %**	**EIGHT CELL EMBRYOS %**	**BLASTOCYSTS %**
Bull 1	Conventional	500	72.2 ± 5.63	59.8 ± 11.03	35.8 ± 6.42
	SexedULTRA-4M	500	75.6 ± 5.73	62.8 ± 13.92	40.4 ± 8.91
Bull 2	Conventional	500	46.2 ± 2.28^a^	26.2 ± 1.92	20.4 ± 2.07^a^
	SexedULTRA-4M	500	51 ± 3.32^b^	29.8 ± 3.63	24.2 ± 2.77^b^
Bull 3	Conventional	500	40.8 ± 2.28	18 ± 1.87	10.4 ± 1.14^a^
	SexedULTRA-4M	500	43 ± 2.55	20.8 ± 2.58	14.2 ± 0.83^b^
Bull 4	Conventional	500	58.6 ± 6.02	36 ± 6.48	24.6 ± 6.35
	SexedULTRA-4M	500	62.8 ± 7.79	43.2 ± 6.34	29.8 ± 6.65

Different superscript letters “a” and “b” in the same column for a given bull represent statistical differences (p <0.05).

There were no significant differences between the CONV and ULTRA-4M groups (P>0.05) for all variables analyzed for Bull 1 and Bull 4. However, for Bull 2 ULTRA-4M there were significantly higher cleavage rates (P=0.029) and blastocysts on day 7 (P< 0.04) for ULTRA-4M compared to CONV. In Bull 3, ULTRA-4M was significantly higher (P < 0.001) for blastocysts on day 7 regarding the CONV (Table 1).

## Discussion

The results of this research regarding semen type show that significantly more embryos were obtained with ULTRA-4M semen than with CONV semen after IVF. Considering the IVP of each bull, in two bulls ULTRA-4M semen produced more embryos than CONV semen, and with the other two bulls there were no significant differences in all the variables analyzed, although ULTRA-4M semen produced more embryos numerically. These data do not coincide with previous reports (Cran et al., 1993, 1995; Lu et al., 1999; Zhang et al., 2003; Lu and Seidel, 2004; Fischer-Brown et al., 2005; Wilson et al., 2005, 2006; Palma et al., 2008), where lower cleavage and blastocyst rates were reported when sexed semen was compared with conventional semen. However, in the previous research the semen used was from the old technology with a lower concentration (2.1 x 10^6^ sperm/straw) compared with the new technology employed in this work ULTRA-4M with a higher concentration (4 x 10^6^ sperm/straw).

The difference in the results in the present research may be due to the fact that in previous research, the sexed semen had lower parameters of motility, viability, and acrosome integrity compared to conventional semen (Palma et al., 2008; Mostek et al., 2020; Steele et al., 2020) and fertilization and blastocysts rates (Cran et al., 1993, 1995; Lu et al., 1999; Merton et al.*,* 1997; Palma et al., 2008; Barceló-Fimbres et al., 2011). The present research supports the results obtained by Gonzalez-Marin et al. (2018), evaluating *in vitro* frozen-thawed ULTRA-4M and CONV semen, had significantly higher percentages of total motility, progressive motility, acrosome integrity at 24 h post-thaw compared with CONV semen.

On the other hand, regarding the DNA fragmentation index, Gonzalez-Marin et al. (2018) reported that frozen thawed ULTRA-4M semen had significantly lower DNA fragmentation at all times evaluated (0, 6, 24, 48, 72h) post-incubation at 37 °C compared to CONV semen. Simões et al. (2013) reported that semen samples with lower susceptibility to oxidative stress had less DNA damage, and semen samples with increased oxidative stress had decreased cleavage rates and were also associated with a greater number of apoptotic cells.

Another reason for the results obtained in this research with ULTRA-4M semen could be due to the fact that semen selection was done with the Mini-Percoll gradient technique, and in previous research the semen selection was done using the standard Percoll gradient technique (Cran et al., 1993, 1995; Lu et al., 1999; Zhang et al., 2003; Lu and Seidel, 2004; Fischer-Brown et al., 2005; Wilson et al., 2005, 2006; Palma et al., 2008). It has been documented that Percoll centrifugation reduced the percentage of sperm exhibiting normal acrosomes and increased the percentage of sperm with damage to the acrosome (Oliveira et al., 2011). Particularly in sexed semen the beneficial effects of Mini-Percoll gradient techniques have been well documented on recovery rate (Ferré et al., 2018), embryo development (Machado et al., 2009; Folchini et al., 2002; Missio et al., 2018) and reducing reactive oxygen species (Folchini et al., 2002). These aforementioned aspects could influence the IVP results obtained in the present research.

## Conclusion

In conclusion, under the conditions of this research the ULTRA-4M and CONV semen produced similar bovine IVP results for some bulls, but when the semen was analyzed with the average of the bulls, ULTRA-4M produced significantly more blastocysts than CONV semen. Notably some bulls within the present study produce more embryos with ULTRA-4M than CONV semen. More research is necessary to evaluate the IVP using ULTRA-4M “X” and “Y” chromosome-bearing semen from different breeds compared to CONV.
