# Erratum: Evaluation of novel SexedULTRA-4M technology for *in vitro* bovine embryo production

**DOI:** 10.1590/1984-3143-AR2022-0037

**Published:** 2022-05-27

Due to desktop publishing error the article “Evaluation of novel SexedULTRA-4M technology for *in vitro* bovine embryo production” (DOI https://doi.org/10.1590/1984-3143-AR2022-0018), published in Anim Reprod., 19(1): e20220018, 2022, was published with an error in the article type.

On page 1, where the text reads:

THEMATIC SECTION: VIII INTERNATIONAL SYMPOSIUM ON ANIMAL BIOLOGY OF REPRODUCTION (ISABR 2020/2021)

It should read:

ORIGINAL ARTICLE

The publisher apologizes for the errors.

